# Lift-off Effect for Capacitive Imaging Sensors

**DOI:** 10.3390/s18124286

**Published:** 2018-12-05

**Authors:** Xiaokang Yin, Chen Li, Zhen Li, Wei Li, Guoming Chen

**Affiliations:** Centre for Offshore Engineering and Safety Technology, China University of Petroleum (East China), Qingdao 266580, China; lichen_upc@163.com (C.L.); lizhenndt@163.com (Z.L.); liwei@upc.edu.cn (W.L.); offshore@126.com (G.C.)

**Keywords:** capacitive imaging, lift-off, non-destructive evaluation, defect discrimination

## Abstract

Capacitive Imaging (CI) sensors are capable of non-destructively detecting both surface and hidden defects in dielectric materials and characterizing conducting surfaces through a relatively thick insulation layer. However, the complex Measurement Sensitivity Distribution (MSD) of CI sensors render the sensor capacitance variation with lift-off highly non-linear, which may lead to misinterpretation of defect indications. This work systematically studied the lift-off effect using both Finite Element (FE) analysis and experimental approaches. Sensor MSD was used as a tool to predict the imaging performance. Normalized Variation Ratio (*NVR*) was introduced and used to characterise sensor responses due to defects for a CI sensor. Both the FE analysis and experiments suggest that the lift-off effect for a CI sensor is specimen type and condition dependent. For a given defect, the *NVR* may vary non-monotonically with increased lift-offs. A case study on a glass-fibre composite/aluminium hybrid structure with multiple artificial defects demonstrated the feasibility of defects discrimination using multiple CI scans with increased lift-offs.

## 1. Introduction

Non-destructive evaluation (NDE) techniques have been widely used in various applications, i.e., crack detection, material degradation evaluation, weld joint diagnosis, etc., to ensure system safety and reliability [1,2]. In recent years, many novel NDE techniques have been investigated to target the ever growing inspection challenges. The Capacitive Imaging (CI) technique, which uses the fringing quasi-static electric field from coplanar electrodes, has emerged as a promising technique for the NDE of glass-fibre composites [3], composite insulators [4], concrete [5], and Corrosion Under Insulation (CUI) [6]. Similar to the eddy current [7,8,9] and many other electromagnetic NDE methods [10], the CI technique is also sensitive to sensor lift-off. Due to the non-linearity of the probing electric field, the measurement sensitivity value of a coplanar CI sensor in each position in the sensing area is heavily position dependent. Previous work indicated that the measurement sensitivity at different positions under a given CI sensor can vary from positive, through zero to negative [11]. It is also found that the measurement sensitivity distribution can be greatly affected by sensor lift-off, leading to very different imaging results. The complex capacitance changes due to lift-off variations, referred to as the lift-off effect, is previously known to be one of the main obstacles for effective CI NDE as it can easily mask defects. Previous knowledge suggested that the measurements using coplanar capacitive sensors should always be taken at a minimal lift-off/in contact with the specimen, as it is believed that increased lift-offs will greatly and monotonically reduce measurement sensitivity. However, thanks to the high precision measurement circuits now available, the latest practical CI measurements do not always agree with this argument.

Extensive efforts, including sensor design and optimization, advanced signal processing techniques, and new inversion methods, have been presented into the reduction of the lift-off effect in the eddy current and related techniques, as it significantly reduces the effectiveness of defect inspection. Fu presented a new approach based on the dynamic trajectories of the fast Fourier transform (FFT) of the received signals for reducing the lift-off effect [12]. Y. He found that differential process can eliminate the lift-off in PEC defect classification in both time the domain and frequency domain [13]. Fan presented a model-based inversion method in terms of lift-off reduction for eddy current characterization of a plate [14] and investigated the behaviors of LOI due to a plate with varying conductivity and thickness in PEC response for elimination of lift-off effect [15]. Ribeiro used the theory of the linear transformer to explain the lift-off effect with special attention to the point of interception phenomenon and presented an apparatus capable of measurement of the thickness of a metallic non-ferromagnetic plate [16]. For capacitive sensors, up to now, little attention has been paid to the lift-off effect in the CI technique. Studies on the lift-off effect [17] and their reduction methods for capacitive sensors, mainly with novel sensor designs [18,19], can only be found sporadically in the literature. It is thus important to carry out a systematic investigation of the lift-off effect and to propose an efficient way to use information arising from varying lift-offs for defect characterisation.

In this work, the concept of the Measurement Sensitivity Distribution (MSD) was introduced and the lift-off effect on the MSD for different types of specimens is discussed in Section 2. This is then followed by analysis on Finite Element (FE) models. The lift-off effect for surface/hidden defects in non-conducting specimens and defects on conducting surfaces through insulation were studied using FE models in Section 3. CI experiments with different lift-offs were also carried out on a glass-fibre composite specimen, and an aluminium specimen. Both surface and hidden artificial defects (machined flat-bottom holes with different depths) were utilized to simulate the commonly seen defect types in practical applications. The experimental results are presented and discussed in Section 4. Discussion and conclusions are then presented in Section 5.

## 2. Measurement Sensitivity Distribution (MSD) and the Lift-Off Effect

### 2.1. The CI sensor and Its MSD

**Figure 1:**
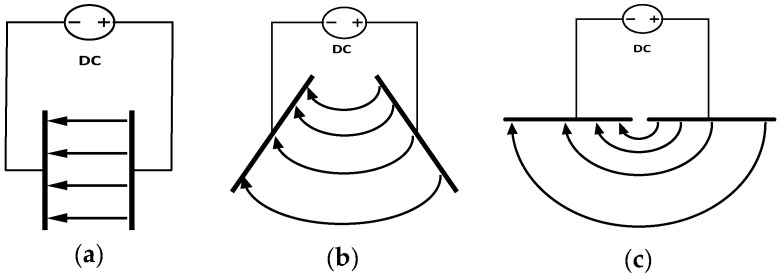
Electric field distribution as two conventional parallel electrodes (**a**) changing from angled electrodes (**b**) into two co-planar electrodes (**c**).

A CI sensor consists of two co-planar electrodes, one being the driving electrode and the other being the sensing electrode. The electric field around two co-planar electrodes is different from the electric field around two conventional parallel electrodes. Figure 1 shows the variation of the electric field distribution with the electrodes changing from parallel to co-planar.

In practical CI scans, the co-planar sensor generates an electric field distribution in the vicinity of the sensor when an AC voltage (usually 10 kHz) is applied to the driving electrode(s). Scanning such an electrode pair across a surface and measuring the resultant change in stored charge for a given voltage creates a map of changes in electrical properties in the probing area, and a capacitive image is thus formed.

**Figure 2:**
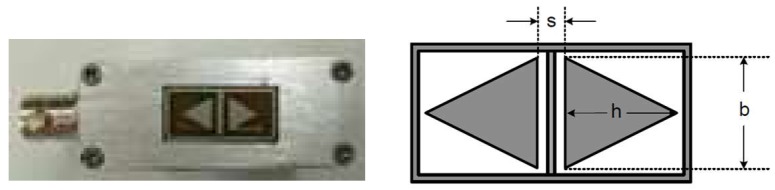
CI sensor with triangular electrodes.

A coplanar CI sensor with back-to-back triangular electrodes, shown in Figure 2, was studied in this work as an example, as this sensor geometry is thought to be able to balance the many trade-offs among the penetration depth, imaging resolution, and signal strength. The CI sensor, which was fabricated on a double-sided printed circuit board (PCB), could be specified by the base (*b*) and height (*h*) of each triangle electrode and the separation distance (*s*) between the parallel edges of the two triangles. In this particular case, the co-planar CI sensor is with *b* = 8 mm, *h* = 8 mm and *s* = 2 mm. The surrounding guard electrodes are 1 mm in width. The thickness of the FR4 substrate of the PCB is 1.5 mm. The backplane of the PCB is coated with metal, which was grounded during CI scans and used as a backplane guard. The whole PCB is embedded into a metallic shell for further shielding.

Due to the non-linearity of the probing field, it is inappropriate to characterise a CI sensor with a single sensitivity value, as the measurement sensitivity value at each point in the sensing area is position dependent and can be described as a measurement sensitivity distribution (MSD). The MSD can also be considered as a 3D point spread function (PSF) in the imaging process and used to predict the imaging performance of the sensor. The measurement sensitivity value *S* at a given position with coordinate (*x*, *y*, *z*) can be calculated using Equation (1):

(1) $$S(x,y,z)=-\vec{\xi D}\cdot \vec{\xi S},$$

where ξD and ξS are the electric fields in the position (*x*, *y*, *z*) when the driving and sensing electrodes are energised with a unit voltage respectively, referred to as reciprocal energization [10]. The measurement sensitivity value *S* at a given point describes how effectively that point is contributing to the charge variation on the sensing electrode.

**Figure 3:**
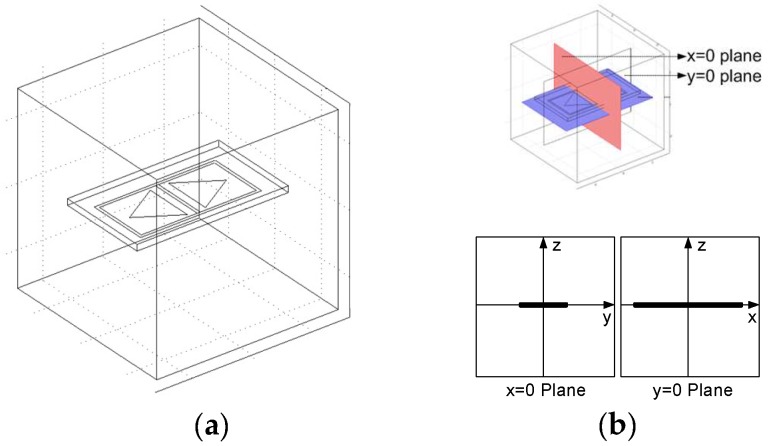
The 3D FE model: (**a**) the computational domain with a CI sensor (**b**) plane coordinate systems for the two kinds of cross sections.

Depending on the position in the sensing area, the angle between ξD and ξS can be less than, equal to or greater than 90 degrees, thus as the inner product of these two vector, the measurement sensitivity value can be positive, zero and negative. To obtain the MSD of the CI sensor shown in Figure 2, a 3D FE model was constructed in COMSOL (AC/DC module), as shown in Figure 3.

The frequency involved in the CI measurement is within the quasi-static range, in which the magnetic field can be considered to be decoupled from the electric field. In addition, the employment of an electrode pair (rather than coils) maximised the effect of the electric field. Therefore, an electrostatic analysis should be adequate to describe the CI measurement and a DC excitation on the driving electrode was used in the FE models. A detailed description on the quasi-static approximation and related governing equations for the FE model has been presented in the authors’ previous work [5,10], and the setup of the model used in this work is described here. A 30 mm × 30 mm × 30 mm block centred at point (*x* = 0, *y* = 0, *z* = 0) was defined to be the computational domain. A 25 mm (*x*-axis) by 15 mm (*y*-axis) rectangle was drawn on the *z* = 0 plane and extruded upwards (positive *z* direction) to be a 2 mm thick plate as the dielectric substrate of the CI sensor, as shown in Figure 3a. In the coordinate system shown in Figure 3b, based on which the results will be presented hereafter, the sensor surface is centered at (*x* = 0, *y* = 0, *z* = 0). *x* = 0 plane is the cross-sectional plane along the short axis of symmetry of the CI sensor, and *y* = 0 plane is the cross-sectional plane along the long axis of symmetry of the CI sensor. The computational domain for studying sensor fields in air only assumed the dielectric constant to be 1. The material of the insulating substrate of the PCB was flame retardant woven glass reinforced epoxy resin (FR-4), and accordingly, the dielectric constant was set to 4.5. Since the electrodes and backplane are very thin copper layers (35 μm), they were treated as boundaries rather than sub-domains. This can reduce the computation complexity to a great extent as very thin sub-domains will require a very fine mesh element and increase the total number of the elements. All sides of the computational domain except the bottom plane (*z* = −15 plane was set to be ‘ground’) have a Neumann boundary condition which can be expressed as:

(2) $$\partial \Phi /\partial \vec{n}=0,$$

where $\vec{n}$ is the direction perpendicular to the surfaces. The conditions on all internal surfaces apart from the metal parts of the sensor were set to ‘continuity’. The electric potential of the driving electrode was set to be 1 V and the electric potential of the sensing electrode was set to be 0 V (Dirichlet boundary condition). Guard electrodes and backplane of the sensor were set to ‘ground’. The ‘triangle mesh’ was used and the number of elements was typically 4,000,000 which was about the maximum limit the PC (2.4 GHz Intel^®^ Xeon^®^ 2 Duo processor, 64.0 G RAM, Gentai, Shanghai, China) could handle. The FE model can be used to predict the electric field distribution under the CI sensor, and to calculate the capacitance under various experimental conditions.

**Figure 4:**
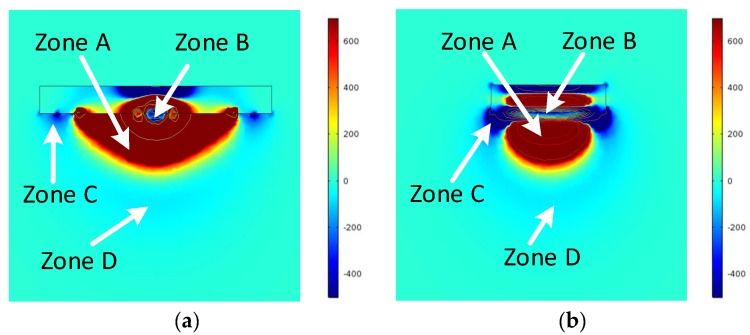
The MSD for the CI sensor shown in Figure 1 (**a**) MSD in Y = 0 plane and (**b**) MSD in X = 0 plane.

To obtain the MSD of the CI sensor, the two triangular electrodes in the FE model were reciprocally energized by a unit voltage and the relative electric field distributions (ξD and ξS) were obtained. Based on Equation (1), the two cross-sections, namely *y* = 0 plan and *x* = 0 plane, of the MSD of the CI sensor shown in Figure 1 can be obtained, as shown in Figure 4.

The MSD under the CI sensor surface contains positive measurement sensitivity distribution region (“Zone A” in Figure 4) and negative measurement sensitivity distribution regions (“Zone B”, “Zone C” and “Zone D” in Figure 4). The positive measurement sensitivity distribution region (“Zone A” in Figure 4a) lies around the driving electrode and the sensing electrode, and has a positive effect on signal strength for detecting defects in both conducting and non-conducting material. The negative measurement sensitivity distribution regions consists of three parts, the first one being between the driving electrode and the sensing electrode (“Zone B” in Figure 4a), the second one being outside the two electrodes (“Zone C” in Figure 4a), and the third one being further way from the sensor surface (“Zone D” in Figure 4a). All parts of the negative measurement sensitivity distribution regions have negative effect on signal strength for detecting defects. The negative sensitivity zones near the sensor surface (“Zone B” and “Zone C” in Figure 4) are caused by grounded guard electrodes in the sensor. Although such guard electrodes bring in complexity in the measurement sensitivity distribution, they are indispensable due to their contributions on shielding stray field and increase penetration depth of the CI sensor [20].

For a given specimen under test, the lift-off effects on the imaging performance are two-folded. Firstly, the whole MSD will be different (as shall be discussed in Section 2.2). Secondly, the relative location of the possible defect in the MSD will be different, leading to very different outputs (as shall be discussed in Section 3).

### 2.2. Analysis on the Lift-Off Effects Based on the Sensor MSD

**Figure 5:**
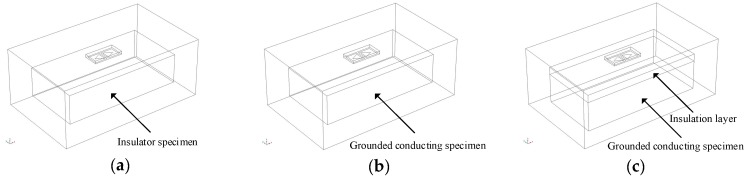
Model geometries for (**a**) Model 1, (**b**) Model 2 and (**c**) Model 3.

3D FE models with three types of specimens under test, which representing the three typical applications, namely insulator (Perspex-Model 1), conductor (aluminium-Model 2) and conductor with insulation layer (Model 3), were constructed to study the lift-off effect. The dimensions of the computational domain for the models are 100 mm × 60 mm × 40 mm, model configurations, apart from different specimen properties, remained the same as the FE model in Section 2.1. The model geometries are shown in Figure 5. The MSDs were calculated and MSDs in the *Y* = 0 plane were plotted.

**Figure 6:**
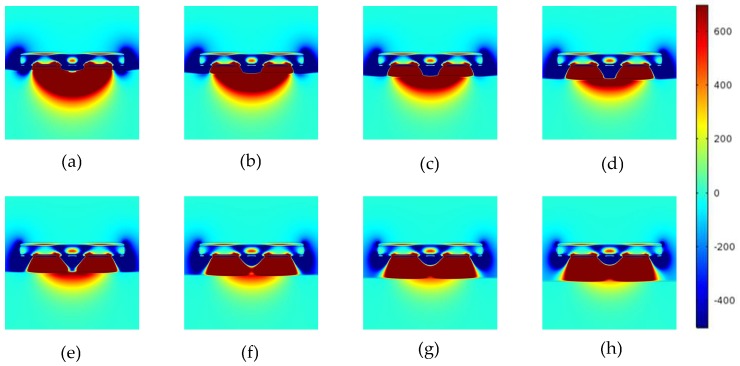
MSD variations with increased lift-offs for a non-conducting specimen. MSDs at (**a**) 0.5 mm, (**b**) 1 mm, (**c**) 1.5 mm, (**d**) 2.0 mm, (**e**) 2.5 mm, (**f**) 3.0 mm, (**g**) 3.5 mm, and (**h**) 4.0 mm lift-offs.

**Figure 7:**
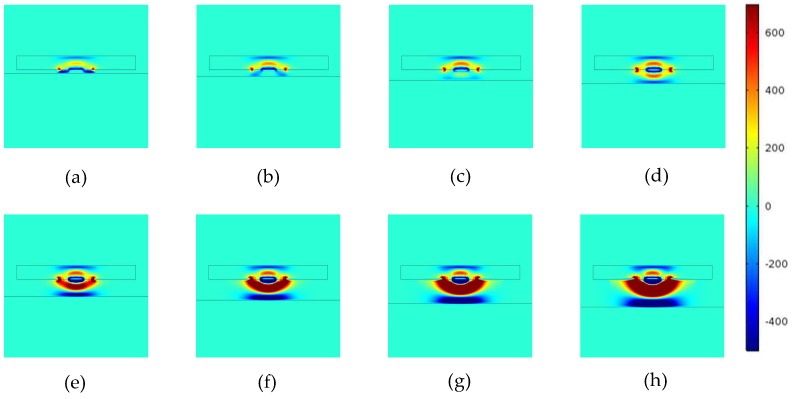
MSD variations with increased lift-offs for a grounded conducting specimen. MSDs at (**a**) 0.5 mm, (**b**) 1 mm, (**c**) 1.5 mm, (**d**) 2.0 mm, (**e**) 2.5 mm, (**f**) 3.0 mm, (**g**) 3.5 mm, and (**h**) 4.0 mm lift-offs.

**Figure 8:**
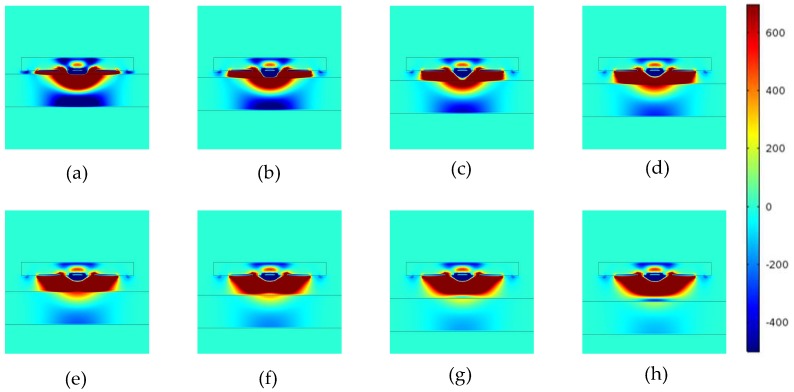
MSD variations with increased lift-offs for a conducting specimen covered by an insulation layer. MSDs at (**a**) 0.5 mm, (**b**) 1 mm, (**c**) 1.5 mm, (**d**) 2.0 mm, (**e**) 2.5 mm, (**f**) 3.0 mm, (**g**) 3.5 mm, and (**h**) 4.0 mm lift-offs.

The non-conducting specimen used in Models 1 is a Perspex (*ℇ* = 4) plate. The MSDs with different lift-offs for Model 1 is shown in Figure 6, the MSDs for Model 2 is shown in Figure 7, and the MSDs for Model 3 is shown in Figure 8. The MSDs are visualized and the influences of the different boundaries with increased lift-offs are demonstrated. For direct comparison, the MSDs are plotted in identical colour range. The MSDs were calculated with the lift-offs being 0.5, 1, 1.5, 2, 2.5, 3, 3.5 and 4 mm for all the three models.

The MSDs for the three cases were all affected by the increased lift-offs. For a non-conducting specimen, as shown in Figure 6, the MSDs will penetrate into the specimen and be similar to the MSD obtained in air. Negative sensitivity values are primarily located at the centre and edges (where the grounded guard electrodes are located) near the sensor surface, and can hardly be found at depth in the specimen.

For a conducting specimen, as shown in Figure 7, the interface act as a “hard boundary” (no electric field line can penetrate into the specimen), preventing the MSDs from penetrating into the specimen. The negative sensitivity value zones are concentrated above the conducting surface even with very small lift-offs.

The conductor with insulation layer case, as shown in Figure 8, is a combination of the former two cases. Due to the presence of the hard boundary, the negative sensitivity zones are concentrated above the conducting surface in the non-conducting layer.

The insulation layer in Model 3 is 5 mm thick, therefore, the MSDs for this case is a similar version for the conductor case, but with even greater lift-offs (the distance between the sensor and metal surface changed from 5.5 mm to 9.5mm). Comparing Figure 7 and Figure 8, the blue colour became darker with increased lift-offs at the beginning and gradually faded at greater lift-offs. This suggest that there might exist an “optimal lift-off” for surface features on conducting specimen, as shall be demonstrated in later sections.

## 3. Finite Element (FE) Analysis on the Lift-Off Effects in Defect Detection

**Figure 9:**
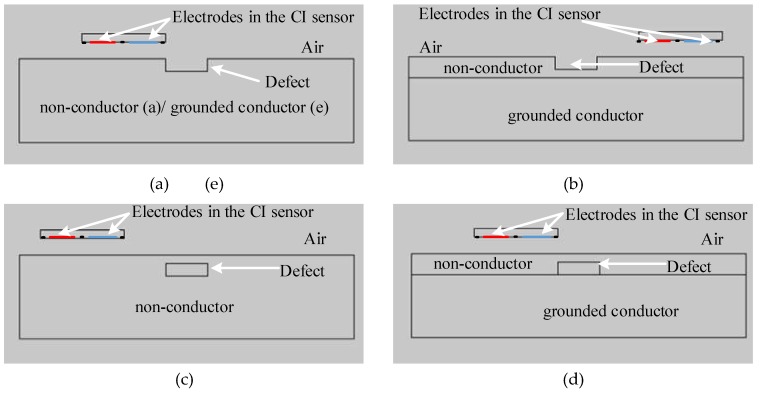
FE Model geometries for (**a**) Model 4, (**b**) Model 5, (**c**) Model 6, (**d**) Model 7 and (**e**) Model 8.

To investigate the lift-off effect on defect detection, artificial defects were introduced into the a specimens under test to simulate surface defects in thick non-conducting material (Model 4), surface defects in a non-conductor with conducting substrate (Model 5), hidden defects in non-conductors without conducting substrates (Model 6), hidden defects in non-conductors with conducting substrates (Model 7), and surface defects on a grounded conductor (Model 8). Simulated scans in 3D FE models require too much computation cost, thus 2D models were used to simulate the CI scans. One of the cross-sections (*Y* = 0 plane) was extracted from the 3D models to become 2D models, in which the bodies (computational domain, PCB substrate, specimens) become surfaces and the surfaces (boundaries and electrodes) become lines. Models 4 to 8 are with the same computational dimensions (140 mm × 40 mm) and CI sensor specifications (20 mm × 2 mm). The defects were 10 mm × 3 mm air voids. The geometries of these models are shown in Figure 9.

The non-conducting material used in these 2D models is Perspex (*ℇ =* 4) and the defects are filled with air (*ℇ =* 1). Simulated line scans in these 2D models were with lift-offs varying from 0.5 mm to 4 mm, unless otherwise stated. To compare the ranges of capacitance variation due to the defect in different line scans, a characteristic value, namely Normalized Variation Ratio (*NVR*) is introduced. The *NVR* can be calculated by: *(C_m_−C_0)_/C*_0_, where *C_m_* is the measured capacitance (in the FE models, *C_m_* is the calculated capacitance), *C_0_* is the capacitance measured/calculated at a defect-free position.

### 3.1. Line Scans for a Surface Defect in a Thick Non-Conductor

**Figure 10:**
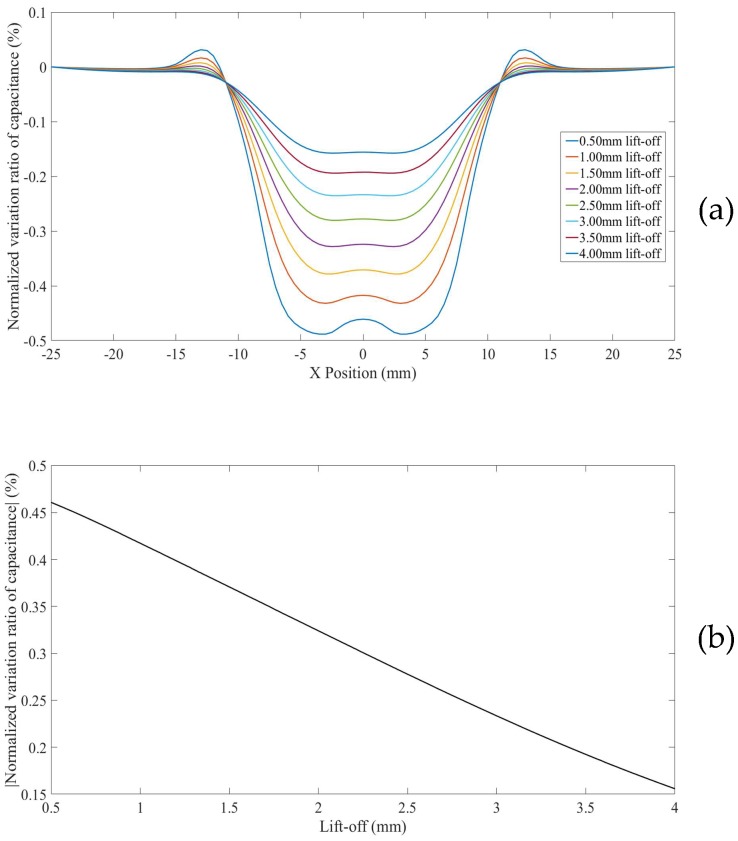
(**a**) *NVR* curves for Model 4 with lift-offs increased from 0.5 mm to 4 mm and (**b**) absolute values of the *NVRs* at the centre point against lift-offs.

For a surface defect in thick non-conducting specimen (Model 4), in which case the influence of grounded plane in the model is minimized, the *NVRs* of capacitance against scan position with the lift-offs increasing from 0.5 mm to 4.0 mm are plotted in Figure 10a. For clarity, the absolute values of the *NVRs* obtained at the central position of the defect for each lift-off were plotted against lift-offs, as shown in Figure 10b. It can be seen from Figure 10a,b, the absolute value of the *NVR* at the centre point decreases monotonously with increasing lift-off. Note that, for small lift-offs (e.g., 0.5 mm) there is a small bump in the centre of the curve due to the negative sensitivity value near the surface as shown in Figure 4.

### 3.2. Line Scans for Surface Defects in Non-Conductors with Conducting Substrates

**Figure 11:**
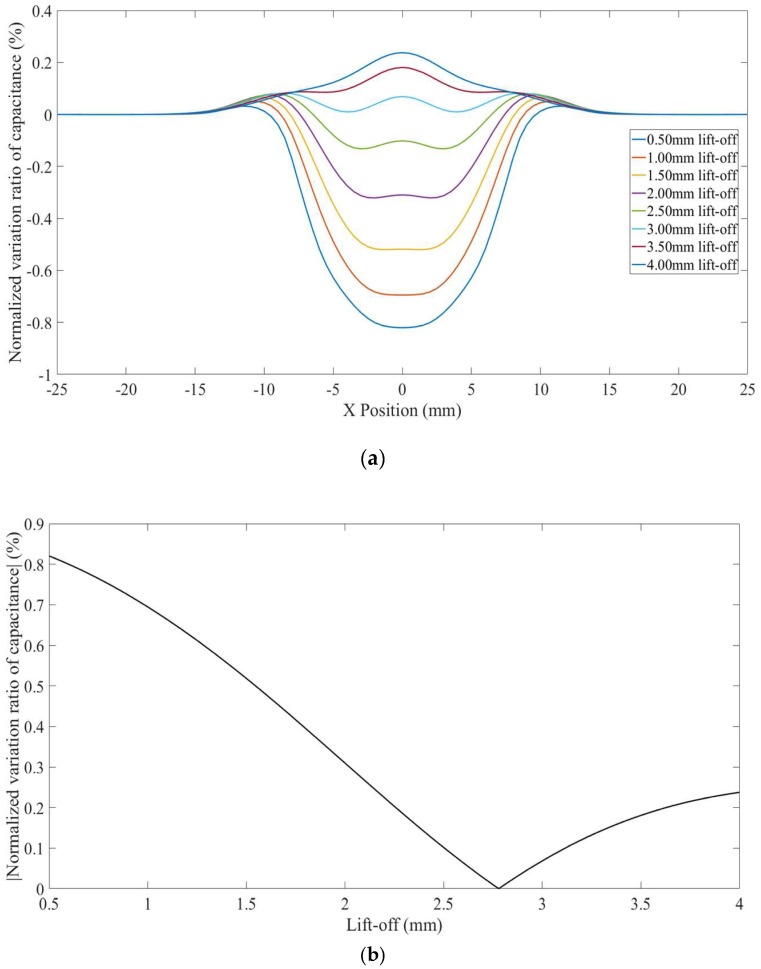
(**a**) *NVR* curves for Model 5 with lift-offs increased from 0.5 mm to 4 mm and (**b**) absolute values of the *NVRs* at the centre point against lift-offs.

For surface defects in thinner non-conducting specimens placed on a conducting substrate (Model 5), the *NVRs* of capacitance against scan position with the lift-offs increasing from 0.5 mm to 4.0 mm are plotted in Figure 11a.

The absolute values of NVRs obtained at the central position of the defect for each lift-off are plotted against lift-offs, as shown in Figure 11b. It can be seen from Figure 11b that, the surface defects appeard as depressed curves for small lift-offs (e.g., 0.5 mm to 3 mm) and bulging curves for big lift-offs (e.g., above 3.5 mm). It can also be noted that, the absolute value of the NVR at the centre point decreases at first, across zero, and then increases slightly with increased lift-offs. This is because the presence of the grounded plane concentrated the negative measurement sensitivity zone above the grounded plane inside the non-conducting specimen (shown in Figure 8). As a result, at bigger lift-offs, the defect (a smaller permittivity zone) is located in a negative measurement sensitivity zone.

### 3.3. Line Scans for Hidden Defects in Thick Non-Conductors

**Figure 12:**
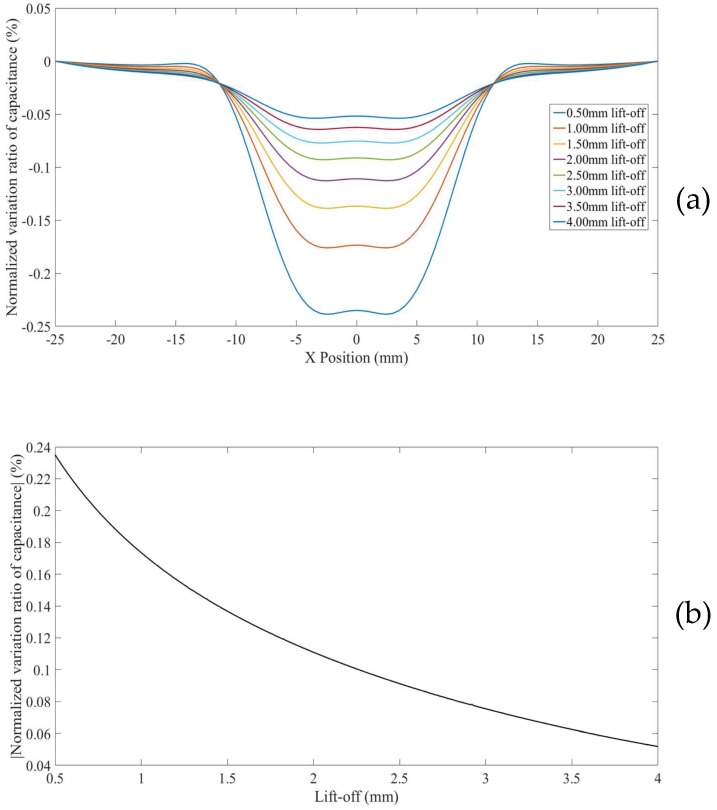
(**a**) *NVR* curves for Model 6 with lift-offs increased from 0.5 mm to 4 mm and (**b**) absolute values of the *NVRs* at the centre point against lift-offs.

Scans with increased lift-offs for hidden defects in thick non-conducting specimens without a conducting substrate (Model 6) were also simulated. The *NVRs* against scan position are plotted in Figure 12a, and the *NVRs* variation at the centre point of each scan against lift-offs is plotted in Figure 12b. It can be seen from Figure 12a,b that the *NVRs* decrease monotonously with increased lift-offs.

### 3.4. Line scans for Hidden Defects in Non-Conductors with Conducting Substrates

**Figure 13:**
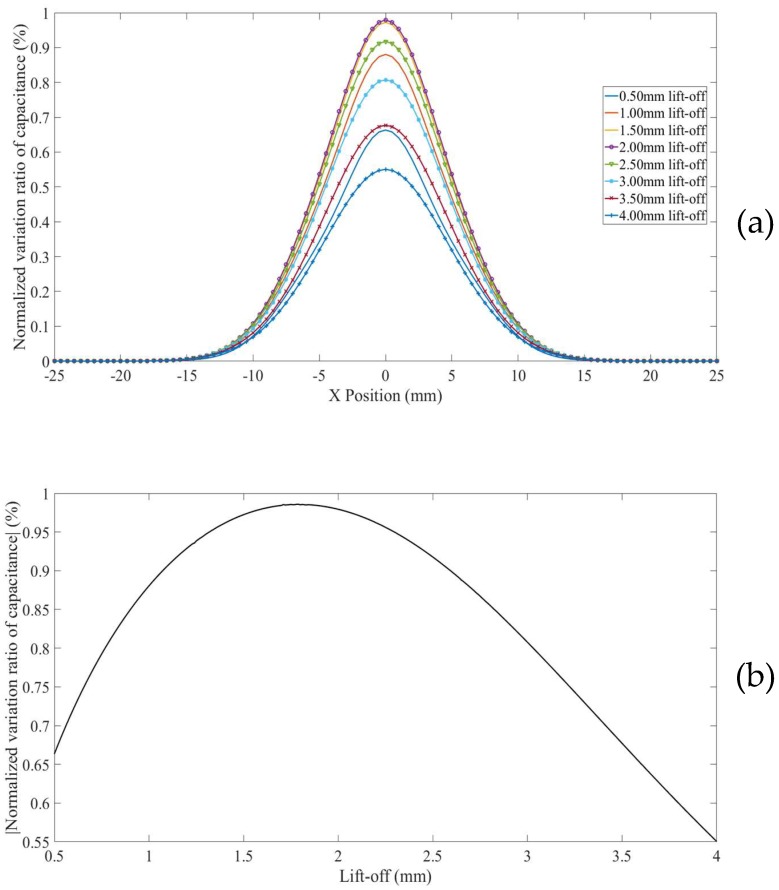
(**a**) *NVR* curves for Model 7 with lift-offs increased from 0.5 mm to 4 mm and (**b**) absolute values of the *NVRs* at the centre point against lift-offs.

Scans with increased lift-offs for hidden defects in thinner non-conductors placed on a grounded conducting substrate (Model 7) were also simulated. Note that, contrary to the case discussed in Section 3.3, the hidden defect appeared as bulging curves, as shown in Figure 13a. This is because the grounded substrate “concentrates” the negative sensitivity value zones, and makes a good part of the defect fall inside the negative sensitivity value zone even at a small lift-off. The *NVR* variation at the centre point of each scan against lift-offs is plotted in Figure 13b. It can be seen from Figure 13b that the *NVR* at the centre point increases at first and then decreases with increased lift-offs. This non-monotonous trend is caused by the negative measurement sensitivity zone in the MSD-with increased lift-off, the negative measurement sensitivity zone takes different portion of the defect. Note that, the buried depths for the defects in Model 6 and Model 7 are the same. However, the defects appeared as opposing variations in the simulated CI scans. This is again because the presence of the grounded plane concentrated the negative measurement sensitivity zone above the grounded plane inside the non-conducting specimen (shown in Figure 8), and made the defect in Model 7 in the negative sensitivity value zone even at small lift-offs.

### 3.5. Line Scans for Surface Defects on Grounded Conductors

**Figure 14:**
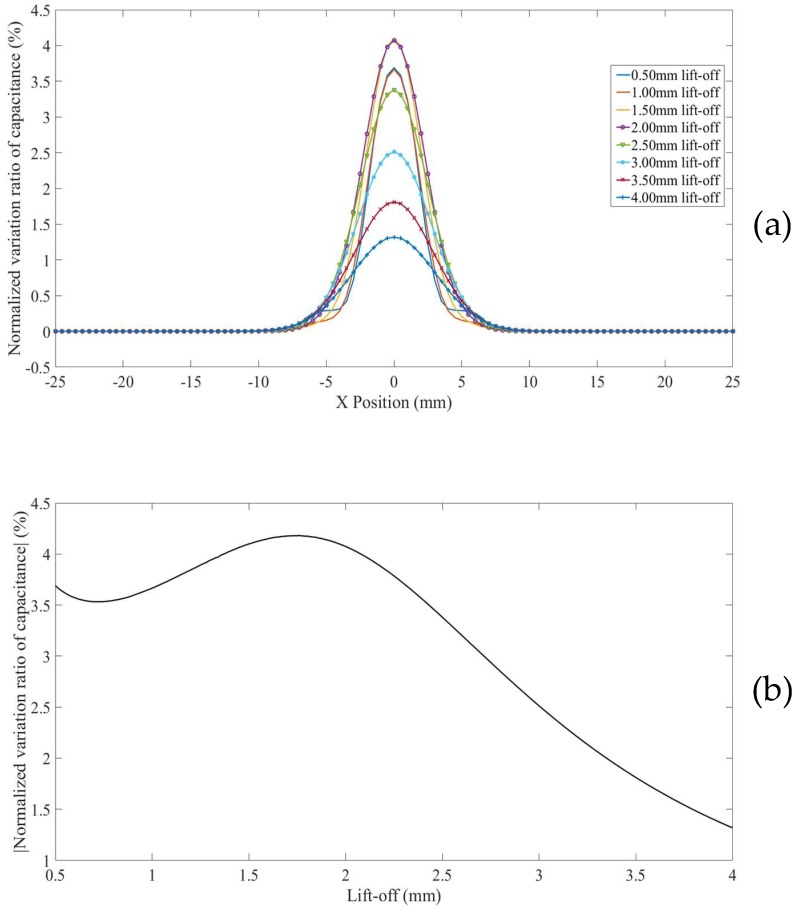
(**a**) *NVR* curves for Model 8 with lift-offs increased from 0.5 mm to 4 mm and (**b**) absolute values of the *NVRs* at the centre point against lift-offs.

For surface defects in conducting specimens, in which case the specimen was explicitly grounded, the *NVRs* of capacitance against scan position with the lift-off increasing from 0.5 mm to 4.0 mm are plotted in Figure 14a.

The *NVRs* obtained at the central position of the defect for each lift-off are plotted against lift-offs, as shown in Figure 14. It can be seen from Figure 14a,b, the *NVR* decreases at first, increases to its local maximum at roughly 1.7 mm lift-off, then decreases with increased lift-offs. This indicates that at a 1.7 mm lift-off, such a CI sensor has an optimal imaging performance for surface defects on conducting specimens. There might be higher *NVR* values at much smaller lift-off (e.g., 0.1 mm, which is not shown in this paper) according to the trend shown in Figure 14b, however, for practical CI scans, minimal lift-off above a conducting surface is not always achievable, for instance, in a metal with a thick insulation case. Note that, 2D models used in the abovementioned five cases assume the model is uniform and infinite along the *X*-axis direction, thus they can only provide approximations of the measured/calculated capacitances and the *NVR* trends. Actual trends of *NVRs* at different lift-offs for different cases need to be revealed by experiments, as shall be shown in Section 4.

## 4. Lift-Off Experiments

**Figure 15:**
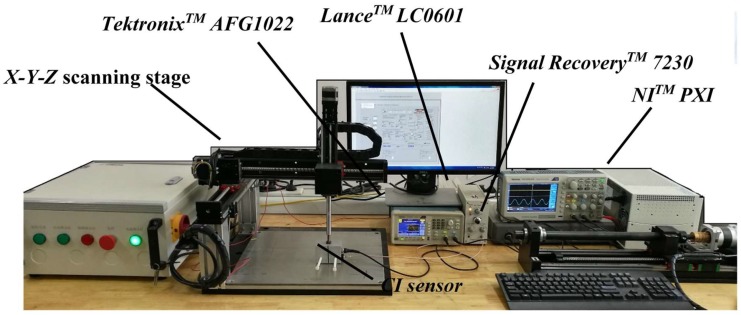
Experimental Setup.

Experiments were designed and carried out to study the lift-off effect for the cases discussed in the previous section. The CI sensor shown in Figure 2 was used in all the experiments in this section. The sensor was part of the basic CI instrumentation. To measure the signal at any particular location, a single frequency AC voltage (10 V pk-pk at 10 kHz) from a signal generator (AFG1022, TektronixTM, OH, USA) was applied as the driving voltage to one of the electrodes in the CI sensor. A LC0601 charge amplifier (Lance^TM^, Qinhuangdao, China) was used to convert the charge signal on the sensing electrode to an AC voltage signal, which could then be recorded if desired. A 7230 lock-in amplifier (Signal Recovery^TM^, TN, USA) which converts the AC voltage signal into a DC voltage proportional to the amplitude of the received AC signal. The DC output was then recorded by the PXI system (NI^TM^, TX, USA). The NI^TM^ PXI system also controlled an *X-Y-Z* scanning stage, which could be used to translate the CI sensor across the sample surface or change the lift-offs. The experimental setup is shown in Figure 15.

**Figure 16:**
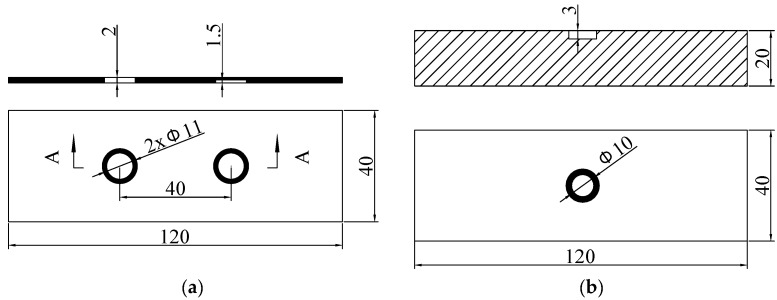
(**a**) Glass-fibre composite board (Specimen I) and (**b**) Aluminium specimens (Specimen II).

The specimens used in the experiments are a 2 mm thick glass-fibre composite boards and a 20 mm thick aluminium board. The glass-fibre composite boards (Specimen I) is with a machined through hole 11 mm in diameter and a 1.5 mm deep machined flat-bottomed hole also 11mm in diameter, as shown in Figure 16a. The aluminium board (Specimen II) is with a flat-bottomed hole 10 mm in diameter and 3 mm in depth (shown in Figure 16b).

**Figure 17:**
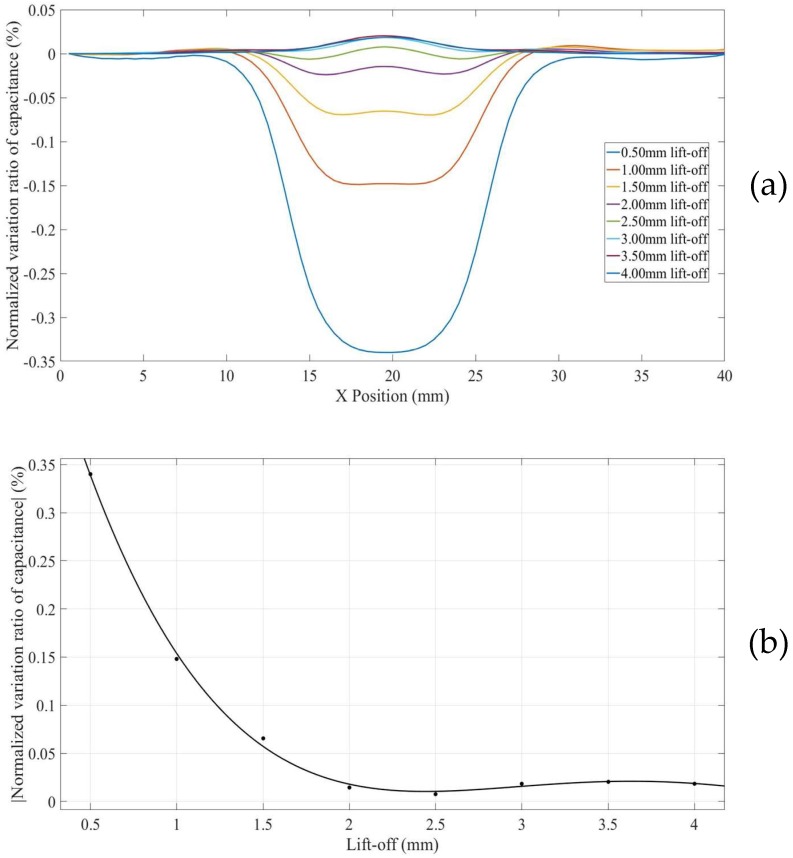
(**a**) *NVR* curves for the through hole on specimen I (placed on a thick insulation plate) with lift-offs increased from 0.5 mm to 4 mm (**b**) absolute values of the *NVRs* at the centre point against lift-offs.

The first set of CI line scans were taken across the through hole on Specimen I, with the specimen placed on top of a 50 mm thick Perspex plate, to study the surface defect in the thick non-conductor case, as discussed in Section 3.1. In this case, the grounded metal plate on the workbench has little influence on the imaging performance. Eight line scans with the lift-off increased from 0.5 mm to 4 mm at a 0.5 mm increment were taken. The *NVRs* of capacitance against scan position with the eight lift-offs are plotted in Figure 17a. For clarity, as in the simulated scans, the absolute values of the *NVRs* obtained at the central position of the defect for each lift-off were plotted against lift-offs, as shown in Figure 17b. It can be seen from Figure 17a that, the surface defect appeared as a depression at smaller lift-offs (i.e., lift-off less than 2.5 mm), while at a bigger lift-off (i.e., lift-off more than 2.5 mm), the defect appear as a bulge. The absolute values of the NVRs obtained at the central position of the defect varies non-monotonically with increased lift-offs, it decreases at small lift-off, reaches its local minimum value at roughly 2.5 mm lift-off, and increases between 2.5 mm and 3.5 mm and slightly decreases again, as shown in Figure 17b. The *NVR* switched its sign, as at a certain lift-off (i.e., roughly 2.5 mm in this case), at this critical lift-off the CI sensor is insensitive to surface defect.

**Figure 18:**
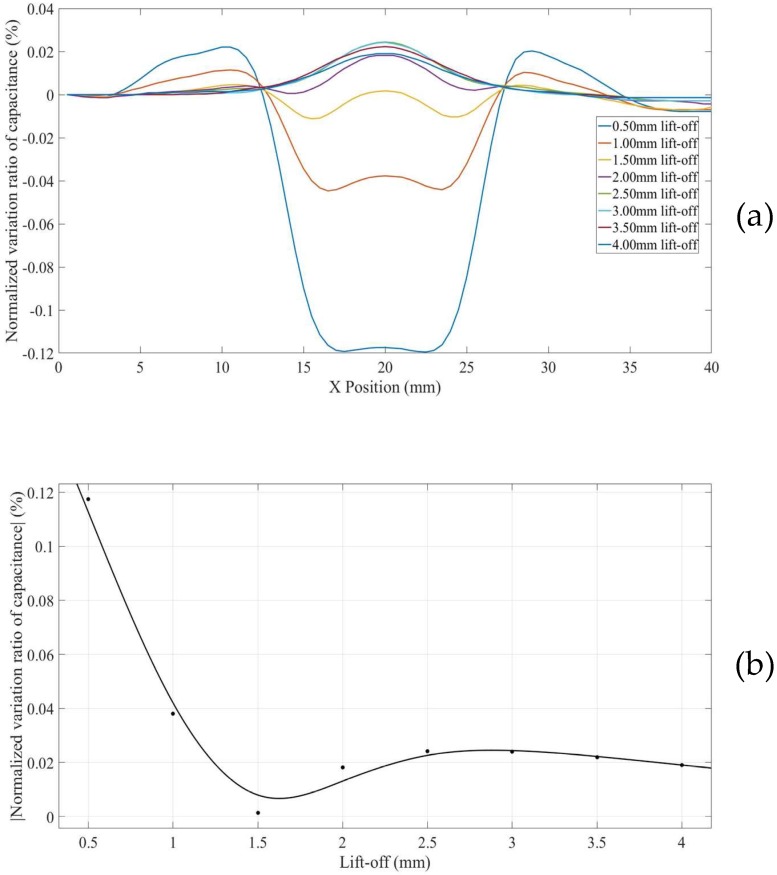
(**a**) *NVR* curves for the through hole on specimen I (placed on grounded conducting plate) with lift-offs increased from 0.5 mm to 4 mm (**b**) absolute values of the *NVRs* at the centre point against lift-offs.

The second set of CI line scans were also taken across the through hole on Specimen I, with the specimen placed on top of a grounded metal plate, to study the surface defect in a non-conductor with a grounded substrate case, as discussed in Section 3.2. Eight line scans with the lift-off increased from 0.5 mm to 4 mm at a 0.5 mm increment were taken. The *NVRs* of capacitance against scan position with the eight lift-offs are plotted in Figure 18a. The absolute values of the NVRs obtained at the central position of the defect for each lift-off were plotted against lift-offs, as shown in Figure 18b. It can be seen from Figure 18a that, the surface defect appeared as a depression at even smaller lift-offs (i.e., lift-off less than 1.5 mm), while at a bigger lift-off (i.e., lift-off more than 1.5 mm), the defect appear as a bulge. The absolute values of the *NVRs* obtained at the central position of the defect varies non-monotonically with increased lift-offs, it decreases at small lift-off, reaches its local minimum value at roughly 1.6 mm lift-off, and increases between 1.6 mm and 3 mm and slightly decreases again, as shown in Figure 18b. Note that in this case the critical lift-off is roughly 1.6 mm.

**Figure 19:**
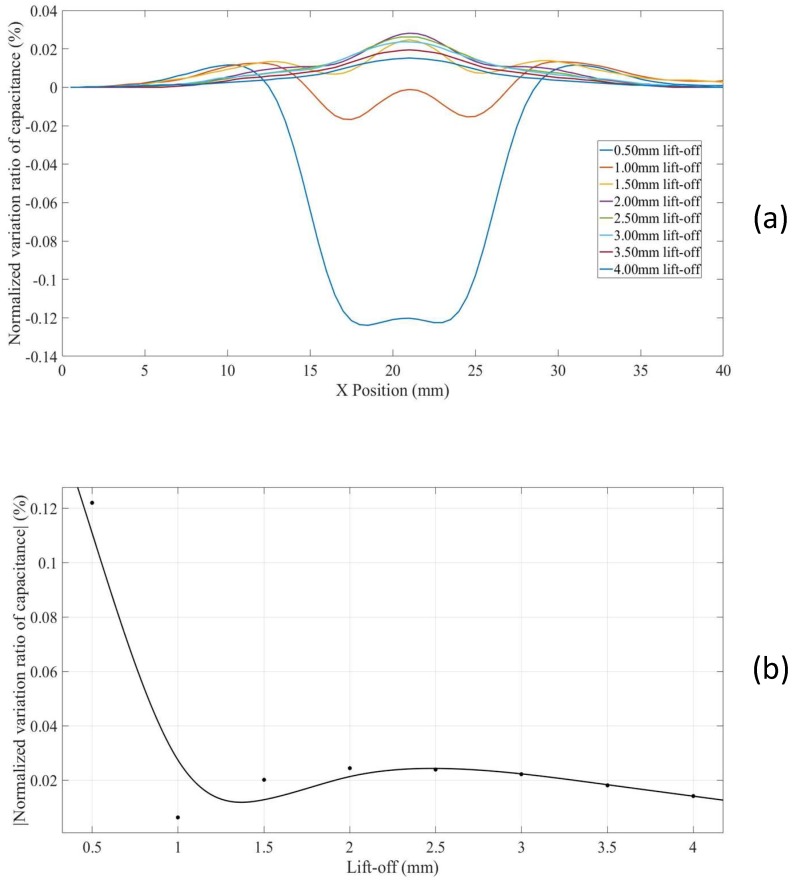
(**a**) *NVR* curves for the hidden 1.5 mm hole on specimen I (placed on a thick insulation plate) with lift-offs increased from 0.5 mm to 4 mm (**b**) absolute values of the *NVRs* at the centre point against lift-offs.

The third set of CI line scans were taken across 1.5 mm deep flat-bottomed hole on Specimen I, with the specimen facing down and placed on a 50 mm thick Perspex plate, to study the hidden defect in a thick non-conductor case, as discussed in Section 3.3. In this case, the grounded metal plate on the workbench has little influence on the imaging performance. Eight line scans with the lift-off increased from 0.5 mm to 4 mm at a 0.5 mm increment were taken. The *NVRs* of capacitance against scan position with the eight lift-offs are plotted in Figure 19a. The absolute values of the *NVRs* obtained at the central position of the defect for each lift-off were plotted against lift-offs, as shown in Figure 19b. It can be seen from Figure 19a that, the hidden defect appeared as a depression at even smaller lift-offs (i.e., lift-off less than 1.3 mm), while at a bigger lift-off (i.e., lift-off more than 1.3 mm), the defect appear as a. The absolute values of the *NVRs* obtained at the central position of the defect varies non-monotonically with increased lift-offs, it decreases at small lift-off, reaches its local minimum value at roughly 1.3 mm lift-off, and increases between 1.3 mm and 3 mm and slightly decreases again, as shown in Figure 19b. Note that in this case, at a lift-off of 1.3 mm, the CI sensor is insensitive to the hidden defect buried 0.5 mm under the surface.

The fourth set of CI line scans were taken across 1.5 mm deep flat-bottomed hole on Specimen I, with the specimen facing down and placed on grounded metal plate, to study the hidden defect in a non-conductor with a grounded substrate case, as discussed in Section 3.4.

**Figure 20:**
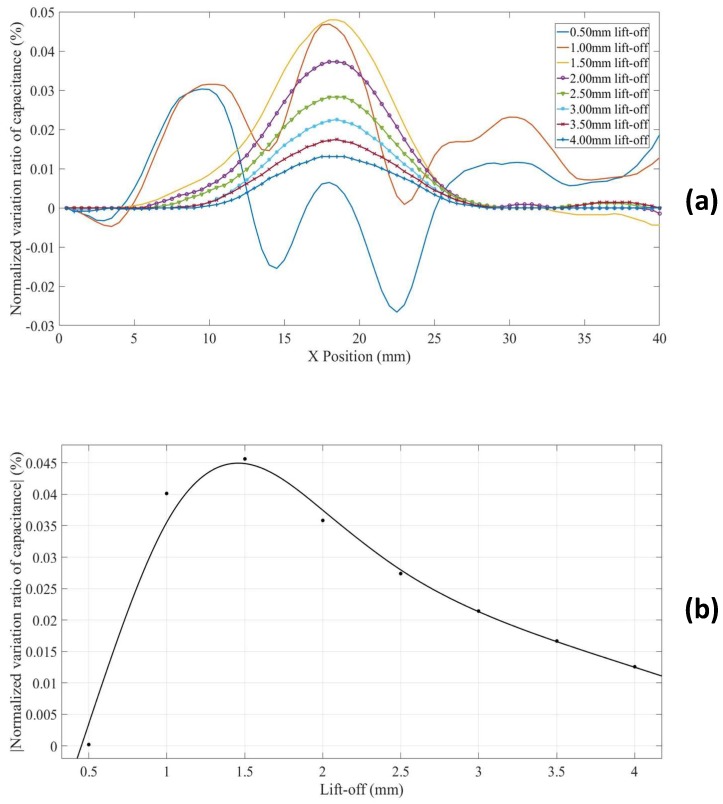
(**a**) *NVR* curves for the hidden 1.5 mm hole on specimen I (placed on a grounded conducting plate) with lift-offs increased from 0.5 mm to 4 mm (**b**) absolute values of the *NVRs* at the centre point against lift-offs.

Eight line scans with the lift-off increased from 0.5 mm to 4 mm at a 0.5 mm increment were taken. The *NVRs* of capacitance against scan position with the eight lift-offs are plotted in Figure 20a. The absolute values of the *NVRs* obtained at the central position of the defect for each lift-off were plotted against lift-offs, as shown in Figure 20b. It can be seen from Figure 19a that, the hidden defect appeared as a depression at 0.5 mm lift-off, and bulges for bigger lift-offs. The absolute values of the *NVRs* obtained at the central position of the defect increase at first and then decreases with increased lift-offs, as shown in Figure 20b. The difference between Figure 19b and Figure 20b suggested that the negative measurement sensitivity value zone of the CI sensor was concentrated in the non-conducting specimen due to the presence of the grounded substrate.

**Figure 21:**
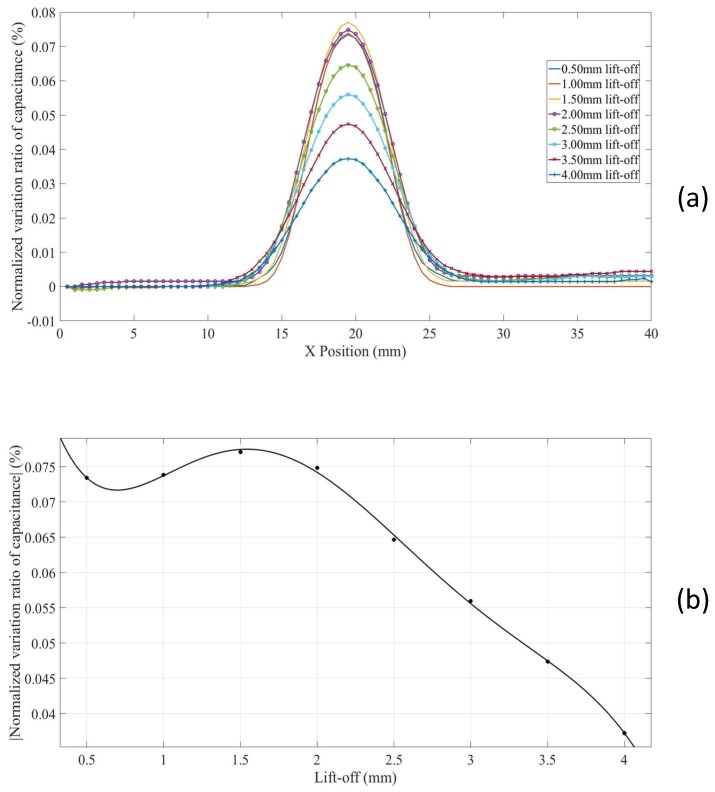
(**a**) *NVR* curves for the surface 3 mm hole on specimen II (grounded) with lift-offs increased from 0.5 mm to 4 mm (**b**) absolute values of the *NVRs* at the centre point against lift-offs.

The fifth set of CI line scans were taken across 3 mm flat-bottomed hole on Specimen II, with the specimen grounded, to study the surface defect on a conducting surface, as discussed in Section 3.5. Eight line scans with the lift-off increased from 0.5 mm to 4 mm at a 0.5 mm increment were taken. The *NVRs* of capacitance against scan position with the eight lift-offs are plotted in Figure 21a. The absolute values of the NVRs obtained at the central position of the defect for each lift-off were plotted against lift-offs, as shown in Figure 21b. It can be seen from Figure 21a that, the surface defect appeared as a bulge for all the lift-offs. The absolute values of the NVRs obtained at the central position of the defect varies non-monotonically with increased lift-offs, as shown in Figure 21b. There exist a local maximum *NVR* value at roughly the 1.5 mm lift-off, suggesting that, such lift-off is an optimal lift-off for surface defect inspection. *NVR* decreases monotonically with increased lift-offs after the so-called optimal lift-off.

**Figure 22:**
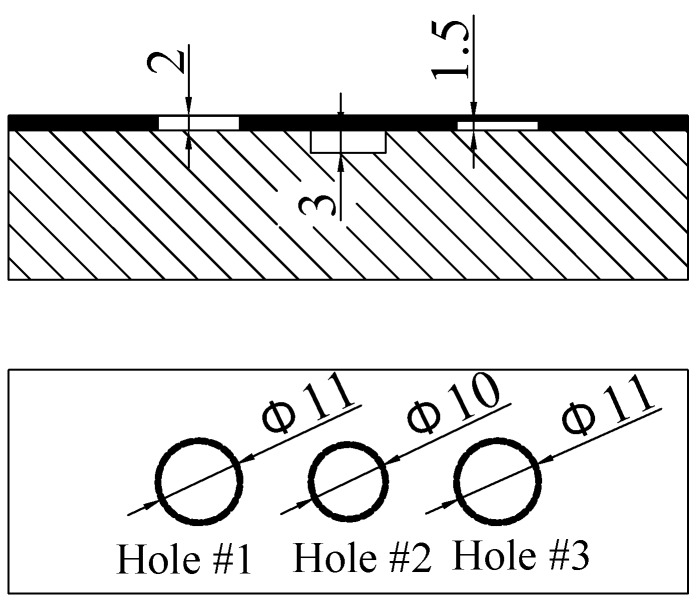
Conducting specimen covered with an insulation layer with three defects.

After CI line scans with various lift-offs for different defects/specimens/experimental conditions were carried out, a hybrid structure with multiple defects was tested, as a case study, to demonstrate how the lift-off effect can help to provide extra information. In this case study, the glass-fibre composite board (Specimen I) was placed on top of the aluminium board (Specimen II), in an attempt to find the different variation trends with increased lift-offs for various types of defects in different materials. The surface hole in the aluminium board was placed between the two holes (the through hole and the 1.5mm hole) in the glass-fibre composite board to simulate different defects in the metal with insulation structure, as shown in Figure 22.

**Figure 23:**
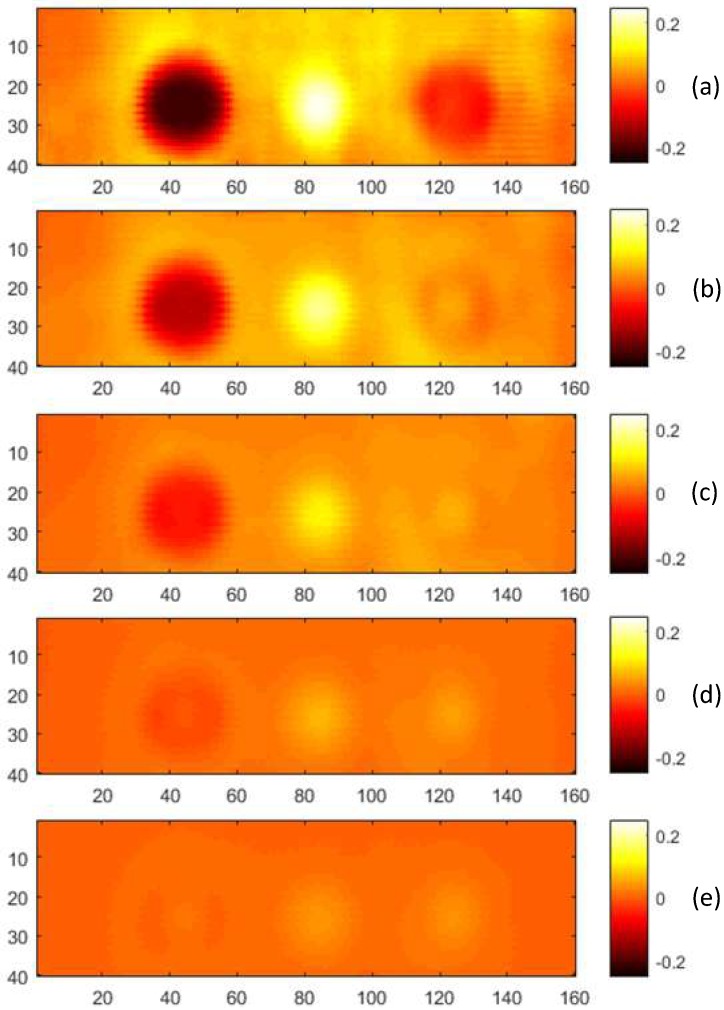
CI scans taken at (**a**) minimal mm lift off, (**b**) 0.5 mm lift-off, (**c**) 1 mm lift-off, (**d**) 1.5 mm lift-off and (**e**) 2 mm lift-off with the aluminium specimen grounded.

The scans were taken with minimal lift-off, 0.5, 1, 1.5 and 2 mm lift-offs. During the CI scans, the aluminium board was grounded. The measured values were also transformed into the *NVRs*, which were used to form capacitive images, as shown in Figure 23. For direct comparison, the images are plotted in the same colour range.

**Figure 24:**
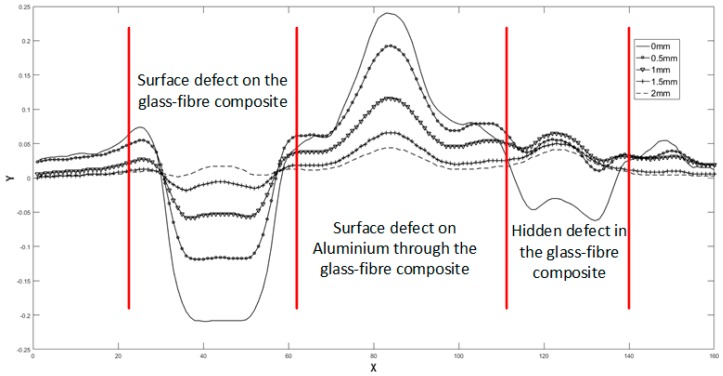
Extracted lines from the capacitive images taken at various lift-offs.

The central line of each scan was extracted and plotted in a single figure (Figure 24).It can be seen from Figure 24 that:

For a minimal lift-off, Hole #1, Hole #3 appeared as depression and Hole 2 appeared as a bulge in the plotted line, while for a 2 mm lift-off, all the three holes appeared as bulges in the plotted line.

For Hole #1, the *NVR* of central position of the hole increased from negative through zero to positive. For Hole #2, the *NVR* of central position of the hole was always positive and decreased with increasing lift-off. For Hole #3, different from the first two holes, the obtained *NVR* lines are crossed (*NVR* @1 mm > *NVR* @0.5 mm > *NVR* @1.5 mm), which indicated that the *NVR* of central position of the hole increased from negative through zero to a local maximum positive value and then decreased slightly.

In this set of experiments, the scans for Hole #1 and Hole #3 are similar to the first set of experiments but with smaller lift-offs. Again, they are in good agreement with the FE models in Section 3.2 and Section 3.4. For the scans for Hole #2, the 2 mm glass-fibre board makes the actual sensor lift-off against metal surface greater than 2 mm, from which the NVRs will decrease monotonically with increased lift-offs, as indicated by the result shown in Figure 21. It can be inferred from Figure 24 that in the cases with defects including surface/hidden voids in the glass-fibre board, and surface metal loss, if a defect appeared as a depression in the resultant image, it must be an air void either on the non-conducting surface or hidden inside. If a defect appeared as a bulge, special attention is required, as it can be any of the three cases. Conclusions can be made by comparing the scans obtained at increased lift-offs, as the trend for the NVRs is unique for each case:

(1) For surface void in the non-conducting board (Hole #1), the NVRs increase monotonously from a negative value, through zero to a small positive value.
(2) For surface metal loss (Hole #2), provided that the insulation cover is thicker than the “optimal lift-off”, the *NVRs* decrease monotonously from a positive value, but never through zero.
(3) For hidden voids in the non-conducting board (Hole #3), the *NVRs* increase from a negative value, through zero, to a relative big positive value and then decrease (the obtained *NVR* lines are crossed).

## 5. Discussion and Conclusions

This paper explores the lift-off effects for coplanar CI sensors based on FE and experimental investigations. MSD is a useful tool to predict imaging performance with varying sensor lift-offs. MSD variations due to non-conducting and conducting boundaries with increased lift-offs can be visualized in the FE models. *NVR* curves against lift-offs for surface/hidden defect in non-conducting specimen and surface defect on conducting specimen were plotted based on simulated CI scans in the FE models. For a non-conducting specimen, with increased lift-off, a defect could change from being in a positive sensitivity value zone to a negative sensitivity value zone. Consequently, the sign of *NVRs* will change at certain lift-offs (referred to as critical lift-off).The presence of conducting substrate will make the so-called critical lift-off smaller, as the grounded plane will push the zero line in the MSD towards the sensor surface and concentrate the negative sensitivity value zone. For surface features on conductors, the measurement sensitivity values are always negative on the conducting surface and surface void will always appear as bulge (high measured values) in the capacitive images. Although, the sign of *NVRs* remain positive, minimal lift-off is not the best case scenario, as there exists an “optimal lift-off”. At the optimal lift-off, a given defect will appear as a greater variation in the capacitive image. This optimal lift-off for a given CI sensor is affected by many factors (i.e., the material type and thickness of the sensor PCB substrate and the material type of the possible insulation layer covering the conducting specimen), and can be determined by FE analysis.

CI experiments were also performed, and the results confirmed that the *NVR* varies non-monotonically with increased lift-offs. The case study on the glass-fibre/aluminium specimen suggested that for an insulator/conductor hybrid structure, to detect and discriminate typical defects (i.e., surface defect on nonconductor, hidden defect in nonconductor and surface defect on insulated conductor), a set of CI scans with increased lift-offs should be carried out. Defect discrimination can then be achieved by analyzing the *NVR* curves against lift-offs, as the *NVR* curve of different defect type has its own characteristic variation trend due to increased lift-offs.

The trends for *NVR* variations with different lift-offs presented in this work is sensor and application dependent, as the MSD for a given CI sensor is heavily affected by the sensor design parameters (including electrode size, shape, spacing and substrate thickness) and the experimental conditions. Such trends can be obtained from FE models and used for defect discrimination. It future, the lift-off effect for conducting specimen with a floating electrical potential and for multi-electrode CI sensors, will be further studied.
